# Cumacea of Greece: a preliminary checklist

**DOI:** 10.3897/BDJ.4.e9287

**Published:** 2016-11-01

**Authors:** Panayota Koulouri, Vasilis Gerovasileiou, Nicolas Bailly

**Affiliations:** ‡ Institute of Marine Biology, Biotechnology and Aquaculture, Hellenic Centre for Marine Research, Heraklion, Greece

**Keywords:** Cumacea, Greece, Aegean Sea, Sea of Crete, Ionian Sea, Eastern Mediterranean, checklist

## Abstract

**Background:**

The first attempt to compile the checklist of Cumacea of Greece was made in the context of the "Greek Biodiversity Database" project (2005-2008) coordinated by the Aristotle University of Thessaloniki. Since then, only scattered information on new elements of the Greek cumacean fauna has been available. The objectives of the present study were to update and cross-check taxonomically all cumacean species records from Greek waters for inaccuracies and omissions according to the recent literature and current taxonomic status.

**New information:**

The updated checklist of Cumacea of Greece, which was built within the framework of the LifeWatch Greece Research Infrastructure (ESFRI) project (2013-2015) coordinated by the Hellenic Centre for Marine Research, comprises 62 species, classified in 24 genera and 6 families. However, a few more records need further cross-checking with the current literature resources.

## Introduction

The most important contributions to the Greek records of the taxonomic group of Cumacea have been made by Reyss (1974) and Bacescu (1982). Later on, Stefanidou (1996), Madurell and Cartes (2003), Mühlenhardt-Siegel (2009), Coll et al. (2010) and Koulouri et al. (2013) have provided new species records and distribution data on the Greek cumaceans. Most cumaceans are hyperbenthic animals, i.e. they are swimming bottom-dependent macrofaunal organisms, performing seasonal or diel vertical migrations into the water column (Brunel et al. 1978). According to Dauvin and Vallet (2006), the hyperbenthos constitutes one of the dominant faunal components of the so called benthic boundary layer (BBL). The BBL is defined as the layer of water adjacent to the seabed and can be extended from a few centimetres to tens of metres, depending, both temporally and spatially, on various physical and biogeochemical processes (Marshall and Merrett 1977). This dynamic zone is characterized by higher concentrations of particles and biomass than the water layer immediately above it. Hyperbenthic sledges are commonly used for sampling these macrofaunal organisms. However, most sledges do not sample the supernatant layer above the seabed in order to avoid contamination of the samples with sediment and therefore there are still technical difficulties in sampling within this particular habitat (Mees and Jones 1997, Eleftheriou 2013, Koulouri et al. 2013).

A first attempt to compile the checklist of Cumacea was made in the context of the "Greek Biodiversity Database" project (2005-2008) coordinated by the Aristotle University of Thessaloniki. The documented occurrence of these marine species in Greek waters was recorded in a database that was set up online in 2010. The reference of Koukouras (2010) was specially created by the World Register of Marine Species (WoRMS)/European Register of Marine Species (ERMS, now part of WoRMS), for the list of these marine species provided by the Greek Biodiversity Database during the European project PESI.

The aim of the present work was to update the checklist of Cumacea of Greek waters. For this purpose, older lists were updated according to the recent literature and current taxonomic status.

## Materials and methods

The checklist of Cumacea of Greece (Suppl. material 1) was built in the context of the LifeWatchGreece (LWG) Research Infrastructure (ESFRI) project, in which the Greek Taxon Information System (GTIS) was established, as an application that has gathered efforts to develop a complete checklist of species recorded from Greek waters (Bailly et al. 2016, this special collection). The general principles for elaborating the Preliminary Checklist are provided by Bailly et al. (2016). The checklist of Cumacea was constructed on the basis of the species records extracted from the dataset of WoRMS/ERMS for marine species. Then, all recent publications were reviewed and the species reported to date have been added to the list. The classification system followed in the present checklist is the one proposed by the Cumacea World Database (Watling 2005).

## Checklists

### Checklist of Cumacea known to occur in Greek waters

#### Cumacea

#### Bodotriidae

#### Bathycuma brevirostre

(Norman, 1879)

#### Bodotria arenosa

Goodsir, 1843

#### Bodotria parvui

Petrescu, 2008

#### Bodotria pulchella

(Sars, 1878)

#### Bodotria scorpioides

(Montagu, 1804)

#### Cumopsis goodsir

(Van Beneden, 1861)

#### Cyclaspis longicaudata

Sars, 1865

#### Eocuma ferox

(Fischer, 1872)

#### Eocuma sarsii

(Kossmann), 1880

#### Iphinoe douniae

Ledoyer, 1965

#### Iphinoe elisae

Băcescu, 1950

#### Iphinoe maeotica

Sowinskyi, 1893

#### Iphinoe rhodaniensis

Ledoyer, 1965

#### Iphinoe serrata

Norman, 1867

#### Iphinoe tenella

Sars, 1878

#### Vaunthompsonia cristata

Bate, 1858

#### Diastylidae

#### Diastylis cornuta

(Boeck, 1864)

#### Diastylis doryphora

Fage, 1940

#### Diastylis jonesi

Reyss, 1972

#### Diastylis neapolitana

Sars, 1879

#### Diastylis rugosa

Sars, 1865

#### Diastyloides bacescoi

Fage, 1940

#### Diastyloides bosphorica

(Băcescu, 1982)

#### Diastyloides carpinei

Bacescu, 1969

#### Diastyloides serratus

(Sars G.O., 1865)

#### Ekleptostylis walkeri

(Calman, 1907)

#### Leptostylis macrura

Sars, 1870

#### Makrokylindrus aegaeus

Reyss, 1974

#### Makrokylindrus longipes

(Sars, 1871)

#### Makrokylindrus spiniventris

Hansen, 1920

#### Vemakylindrus charcoti

(Reyss, 1974)

#### Vemakylindrus doryphora

(Fage, 1940)

#### Lampropidae

#### Hemilamprops normani

Bonnier, 1896

#### Platysympus typicus

(Sars, 1870)

#### Leuconidae

#### Eudorella nana

Sars, 1879

#### Eudorella truncatula

(Bate, 1856)

#### Leucon affinis

Fage, 1951

#### Leucon longirostris

Sars, 1871

#### Leucon macrorhinus

Fage, 1951

#### Leucon mediterraneus

Sars, 1878

#### Leucon siphonatus

Calman, 1905

#### Nannastacidae

#### Campylaspis alba

Hansen, 1920

#### Campylaspis glabra

Sars, 1878

#### Campylaspis horridoides

Stephensen, 1915

#### Campylaspis legendrei

Fage, 1951

#### Campylaspis macrophthalma

Sars, 1878

#### Campylaspis rostrata

Calman, 1905

#### Campylaspis sulcata

Sars, 1870

#### Campylaspis verrucosa

Sars, 1866

#### Campylaspis vitrea

Calman, 1906

#### Cumella limicola

Sars, 1879

#### Cumella pygmaea

G.O. Sars, 1865

#### Cumellopsis puritani

Calman, 1906

#### Nannastacus longirostris

G.O. Sars, 1879

#### Nannastacus turcicus

Băcescu, 1982

#### Nannastacus unguiculatus

(Bate, 1859)

#### Procampylaspis armata

Bonnier, 1896

#### Procampylaspis bacescoi

Reyss & Soyer, 1966

#### Procampylaspis bonnieri

Calman, 1906

#### Styloptocuma gracillimum

(Calman, 1905)

#### Pseudocumatidae

#### Pseudocuma longicorne

(Bate, 1858)

#### Pseudocuma simile

G.O. Sars, 1900

## Discussion

A total of 62 species, classified to 24 genera and 6 families, constitute the updated checklist of Cumacea of Greece. A number of issues aroused during the thorough examination of the checklist of Cumacea of Greece derived from the dataset of WoRMS/ERMS for marine species (Koukouras 2010). According to Coll et al. (2010), only two records of *Iphinoe trispinosa* (Goodsir, 1843) from the Mediterranean Sea exist. However, according to the revision of the genus *Iphinoe* by Ledoyer (1965), there are two more records, one from the North Aegean Sea (Stefanidou 1996) and another from the Gulf of Tunis (Ayari and Afli 2003). Moreover, the presence of two cumacean species recorded by Mühlenhardt-Siegel (2009) as Campylaspis cf. paeneglabra Stebbing, 1912 and Leptostylis cf. bacescoi Reyss, 1972 in the Mediterranean Sea still remains doubtful and should be confirmed. Therefore, further research is needed for the verification of the presence of these three species in the Greek waters. It is also worth mentioning that 27 cumacean species have been reported from the Turkish territorial waters of the Aegean Sea (Katagan 1983, Katagan 1985), while recently the new species *Bodotria parvui* was described by Petrescu (2008) from the same area. Though several species reported from the Turkish coasts of the Aegean have not yet been found in Greek territorial waters, they are expected to be recorded as elements of the Greek fauna in the future.

## Supplementary Material

Supplementary material 1 Checklist of Cuma﻿cea (Arthropoda: Malacostraca) of Greece Data type: Checklist
File: oo_97964.xls Panayota Koulouri, Vasilis Gerovasileiou, Nicolas Bailly

XML Treatment for Cumacea

XML Treatment for Bodotriidae

XML Treatment for Bathycuma brevirostre

XML Treatment for Bodotria arenosa

XML Treatment for Bodotria parvui

XML Treatment for Bodotria pulchella

XML Treatment for Bodotria scorpioides

XML Treatment for Cumopsis goodsir

XML Treatment for Cyclaspis longicaudata

XML Treatment for Eocuma ferox

XML Treatment for Eocuma sarsii

XML Treatment for Iphinoe douniae

XML Treatment for Iphinoe elisae

XML Treatment for Iphinoe maeotica

XML Treatment for Iphinoe rhodaniensis

XML Treatment for Iphinoe serrata

XML Treatment for Iphinoe tenella

XML Treatment for Vaunthompsonia cristata

XML Treatment for Diastylidae

XML Treatment for Diastylis cornuta

XML Treatment for Diastylis doryphora

XML Treatment for Diastylis jonesi

XML Treatment for Diastylis neapolitana

XML Treatment for Diastylis rugosa

XML Treatment for Diastyloides bacescoi

XML Treatment for Diastyloides bosphorica

XML Treatment for Diastyloides carpinei

XML Treatment for Diastyloides serratus

XML Treatment for Ekleptostylis walkeri

XML Treatment for Leptostylis macrura

XML Treatment for Makrokylindrus aegaeus

XML Treatment for Makrokylindrus longipes

XML Treatment for Makrokylindrus spiniventris

XML Treatment for Vemakylindrus charcoti

XML Treatment for Vemakylindrus doryphora

XML Treatment for Lampropidae

XML Treatment for Hemilamprops normani

XML Treatment for Platysympus typicus

XML Treatment for Leuconidae

XML Treatment for Eudorella nana

XML Treatment for Eudorella truncatula

XML Treatment for Leucon affinis

XML Treatment for Leucon longirostris

XML Treatment for Leucon macrorhinus

XML Treatment for Leucon mediterraneus

XML Treatment for Leucon siphonatus

XML Treatment for Nannastacidae

XML Treatment for Campylaspis alba

XML Treatment for Campylaspis glabra

XML Treatment for Campylaspis horridoides

XML Treatment for Campylaspis legendrei

XML Treatment for Campylaspis macrophthalma

XML Treatment for Campylaspis rostrata

XML Treatment for Campylaspis sulcata

XML Treatment for Campylaspis verrucosa

XML Treatment for Campylaspis vitrea

XML Treatment for Cumella limicola

XML Treatment for Cumella pygmaea

XML Treatment for Cumellopsis puritani

XML Treatment for Nannastacus longirostris

XML Treatment for Nannastacus turcicus

XML Treatment for Nannastacus unguiculatus

XML Treatment for Procampylaspis armata

XML Treatment for Procampylaspis bacescoi

XML Treatment for Procampylaspis bonnieri

XML Treatment for Styloptocuma gracillimum

XML Treatment for Pseudocumatidae

XML Treatment for Pseudocuma longicorne

XML Treatment for Pseudocuma simile
